# Use of Autochthonous *Lactiplantibacillus plantarum* Strains to Produce Fermented Fish Products

**DOI:** 10.3389/fmicb.2020.615904

**Published:** 2020-12-03

**Authors:** Barbara Speranza, Angela Racioppo, Daniela Campaniello, Clelia Altieri, Milena Sinigaglia, Maria Rosaria Corbo, Antonio Bevilacqua

**Affiliations:** Department of Agriculture, Food, Natural Resources and Engineering (DAFNE), University of Foggia, Foggia, Italy

**Keywords:** starter, fermentation, fish, DoE, autochthonous bacteria

## Abstract

The present research was aimed to the optimization of the production of a fish fermented *salami-like* product using autochthonous *Lactiplantibacillus plantarum* starters. The activity was performed through two phases: (1) Optimization of fermented fish product composition by using a 2^k-p^ Fractional Factorial Design: the variables tested were nitrites (0–150 ppm), salt (2.5–7.5%), sucrose (0–4%), white pepper (0–0.10%), and fermentation temperature (10–30°C); (2) Product realization and evaluation of its microbiological profile [aerobic microbiota (APC), *Pseudomonadaceae* (PSE), *Enterobacteriaceae* (E), and lactic acid bacteria (LAB) populations], chemico-physical parameters (pH and a_w_), and sensorial quality (odor, texture, color, and overall acceptability) during its storage at 4°C for 21 days. In the first step, the fish pulp was mixed with the appropriate amounts of ingredients, according to the experimental design; each batch was individually inoculated with the studied starter (*L. plantarum* 11, *L. plantarum* 69, and *L. plantarum* DSM1055) at 10^7^ cfu/g and incubated at 10, 20, or 30°C for 7 days. The lowest fermentation time (time to reach pH 4.4) was obtained with 4% sucrose, 100 ppm nitrite and a process temperature of 30°C. In the second step, *salami-like* were produced according to the individuated formulation and inoculated with the studied starters (10^7^ cfu/g); the fish mixture was stuffed into a natural casing and left to ferment at 30°C for 7 days. The use of the selected strains not only assured a correct fermentation but reduced the process time at only 2 days; during refrigerated storage, a good microbiological, chemico-physical and sensorial quality of the final product was recorded for at least 21 days.

## Introduction

The use of starters in food industry is a precious mean to improve both process efficiency and product quality (Corbo et al., 2016). In the last decade, the use of autochthonous microbial strains as specific starter cultures has been suggested: namely, the most robust strains are isolated from a food and studied as potential starter for the same matrix (Corbo et al., 2016; Speranza et al., 2017).

As mainly observed for meat and dairy fermented products, lactic acid bacteria (LAB) seem to give the best performances also as fish starters by significantly improving the products’ quality, accelerating the acidification, and controlling the growth of spoilage bacteria and pathogens. However, while the studies about starter cultures for dairy products, wineries, sausages, and vegetables are very broad in literature, the starter to be used for fish fermentation are still rare and the suggested technologies are few: consequently, today there are no specific starters for fermented fish products.

Once, fermented fish products were mainly diffuse in east and southeast Asia, but more recently, with globalization and market opening, some products are being produced elsewhere or exported from Oriental countries to Europe, North America, and Africa (Adams, 2009); some examples are *Hákarl*, *Surströmming*, and *Rakfisk* diffuse in northern Europe (Jensen et al., 2012; Osimani et al., 2019) or *lanhouin* and *momoni* in West Africa (Anihouvi et al., 2007). Other well-known fermented fish products are *Suan yu* and fish sauces in China, *Plaa-som* and *Nam-pla* in Thailand, *fish nukazuke* and *narezushi* in Japan (Kanno et al., 2012). Due to the not homogeneous and complex nature of fish, the high diversity of fish species fermented, various additives used, and different processes applied (Kose and Hall, 2011), the use of allochthonous commercial starters is not advisable, while the use of autochthonous starters is still under-explored.

A number of researchers have proposed strains of the genera *Tetragenococcus*, *Pediococcus*, *Lactobacillus* and related genera, and *Staphylococcus* for several types of fish fermented products (Yongsawatdigul et al., 2007; Udomsil et al., 2010; Natteewan et al., 2011; Speranza et al., 2015, 2017; Zeng et al., 2016; Liao et al., 2018). Some studies highlighted that the addition of starter cultures shortened fermentation time (FT): for example, Yongsawatdigul et al. (2007) found that *Virgibacillus* sp. used for Thai fish sauce fermentation was able to shorten process time to 4 months. Other researchers found an improvement in the quality of traditional Chinese fermented fish using both *Lactiplantibacillus plantarum* and *Saccharomyces cerevisiae* (Liao et al., 2018) or *Tetragenococcus halophilus* (Udomsil et al., 2010).

In 2017, Speranza et al. (2017) proposed two autochthonous strains of *L. plantarum* as potentially multifunctional starters for fish fermented sausage production, since they gave promising results in preliminary assays by both reducing the FT and guaranteeing a good quality of the fish product. In addition, they also showed potential probiotic properties, such as a good low pH tolerance and promising cell adherence properties.

Therefore, the aim of this research was the optimization of the production of a fish fermented *salami-like* product using autochthonous *L. plantarum* starters. The activity was performed through two main phases:

1. Optimization of fermented fish product composition by using a 2^k-p^ Fractional Factorial Design: the variables tested were nitrites (0–150 ppm), salt (2.5–7.5%), sucrose (0–4%), white pepper (0–0.10%), and fermentation temperature (10–30°C).
2. Product realization and evaluation of its microbiological profile [aerobic microflora, *Pseudomonadaceae* (PSE), *Enterobacteriaceae* (E), and LAB populations], chemico-physical parameters (pH and a_w_), and sensorial quality (odor, texture, color, and overall acceptability) during its storage at 4°C for 3 weeks.

## Materials and Methods

### Optimization of Fermented Fish Product Composition

#### Strains

Three strains were used as potential starters:

Two autochthonous strains labeled with a numeric code (11–69), isolated from intestinal microbiota of sea bream (*Sparus aurata*) and identified at species level as *L. plantarum* (Speranza et al., 2017).

One allochthonous commercial strain, *L. plantarum* DSM 1055, isolated from bread dough and purchased from DSMZ (Deutsche Sammlung von Mikroorganismen und Zellkulturen GmbH, Germania).

The strains were stored at −20°C in de Man Rogosa and Sharpe broth (MRS, Oxoid, Milan, Italy) added with sterile glycerol (J.T. Baker, Milan, Italy); before each assay, they were revitalized in MRS broth incubated at 37°C for 24 h.

#### Design

The optimization of fermented fish product composition was obtained by using a 2^k-p^ Fractional Factorial Design. The variables tested were: concentrations of nitrites, salt, sucrose, white pepper, and fermentation temperature. Table 1 shows the coded levels of each independent variable jointly to the eight combinations tested during the experiment. A control sample, i.e., a further combination in which the variables tested were set to level 0, was also reported. Three factorial designs were set up, one for each starter tested.

**Table 1:** Coded levels and combinations used in the 2^k-p^ Fractional Factorial Design.

Coded level	Nitrites (ppm)	NaCl (%, w/w)	Temperature (°C)	Sucrose (%, w/w)	White pepper (%, w/w)
−1	0	2.5	10	0	0
0	75	5	20	2	0.05
1	150	7.5	30	4	0.10
Combinations
A	0	7.5	30	0	0
B	150	7.5	30	4	0.1
C	150	2.5	30	0	0.1
D	150	2.5	10	0	0
E	150	7.5	10	4	0
F	0	7.5	10	0	0.1
G	0	2.5	30	4	0
H	0	2.5	10	4	0.1
Control	75	5	20	2	0.05

#### Samples Preparation

The optimization was performed by using specimens of croaker (*Argyrosomus regius*; Lepore Mare, Fasano, Brindisi, Italy). The fish was gutted, eviscerated, deboned, filleted, and washed in running tap water; then, the drained fillets were passed through a strainer (Meat Strainer Mod. MMG22, Electrolux, Rimini, Italy). Aliquots of fish pulp (20 g) were mixed with the appropriate amount of NaCl, NaNO_2_, sucrose, and white pepper according to the Fractional Design reported in Table 1. Then, inocula were performed by adding each selected strain (*L. plantarum* 11, 69, or 1055) at a final level of 10^7^ cfu/g. Not inoculated samples were used as negative controls. All samples were incubated at the temperature planned by the experimental design for 7 days, during which the pH decrease was monitored by a pH electrode 50*50T CRISON (Crison Instruments, Barcelona, Spain). The experiments were performed over three independent batches, i.e., for each combination three independent samples were analyzed.

### Product Realization

#### Fish Sausages Preparation

Croaker pulp was obtained as described above. Then, it was mixed with (in g/kg): NaCl, 40; NaNO_2_, 0.10; sucrose, 40; white pepper, 10. Strains (11, 69, and 1,055) were inoculated at 10^7^ cfu/g. A control batch was also similarly prepared and not inoculated. After mixing, samples were mechanically stuffed into collagen casings (28 mm diameter) and incubated at 30°C for 7 days. During fermentation, the pH was evaluated by immersing the probe approximately 1–2 cm below the casings. After 7 days, the samples were stored 4°C for 21 days, during which the changes in pH, a_w_, aerobic microbiota (APC), PSE, E, and LAB populations were measured, as described in Speranza et al. (2017).

During the storage, sensorial quality was also assessed by 15 panelists aging between 25 and 50 years [students and researchers of the Department of Agriculture, Food, Natural resources and Engineering (DAFNE), University of Foggia]. Using a scale ranging from 0 to 5 (where 0 was very poor and 5 was excellent; the acceptability threshold was set to 2), the sensorial overall quality of the fermented sausages was evaluated by estimating color, odor, and texture attributes (Speranza et al., 2017). The experiments were performed in triplicate as reported above.

### Statistics

The pH data obtained in the first phase were modeled through the Weibull equation, as modified by Mafart et al. (2002):

$\mathrm{pH}={\mathrm{pH}}_{0}-{\left(t/\Delta \right)}^{p}$

where, pH and pH_0_ are the pH throughout the time and the initial pH, respectively; Δ, the first reduction time (days), i.e., the time to attain a reduction of 1 unit in cell count; *p*, the shape parameter (*p* < 1 upward curve; *p* > 1 downward curve).

Using the fitting parameters of the Weibull equation, the FT (defined as the time in days to attain a pH of 4.4) was calculated:

$\mathrm{FT}=\delta \;\left(N_{0}-\mathrm{Lc}\right)1/p$

Where *L_c_* is the critical limit (4.4) and it could be considered a “safe pH” (Riebroy et al., 2008).

Fermentation time was used as dependent variable for a multiple regression analysis; concentrations of nitrites, salt, sucrose, white pepper, and fermentation temperature were the independent variables. The modeling was performed through the software Statistica for Windows version 7.0 (Statsoft, Tulsa, Okhla). Significant differences amongst combinations for each fitting parameter were pointed out through one-way ANOVA and Tukey’s test (*p* < 0.05).

Sensory scores were analyzed through the Krustal-Wallis test (*p* < 0.05).

## Results

The first phase was performed to individuate not only the optimal formulation of the fermented product, in terms of salt, sucrose, nitrates and spices concentrations, but also the fermentation temperature, in order to ensure a rapid acidification and guarantee the success of the transformation.

The optimization was achieved through the use of three different 2^k-p^ factorial designs, one for each of the tested targets: two autochthonous strains (*L. plantarum* 11–69) isolated from fish intestine (Speranza et al., 2017) and one allochthonous commercial strain (*L. plantarum* 1055).

For each combination studied, the pH data were fitted through the Weibull model (primary model): Table 2 shows the fitting parameters obtained, i.e., *Δ* (time to reduce the pH by 1 unit) and *p* (the shape parameter), for each kinetics of acidification monitored. The initial pH was 6.20–6.50 and there was generally a gradual acidification over time, more or less vigorous depending on the combination considered. For example, for *L. plantarum* 69, the time to reduce pH by 1 ranged from a minimum of 0.52 days (±0.31) of the combination G (2.5% salt, 4% sucrose, without nitrites and pepper, fermentation temperature of 30°C) to a maximum of 23.05 days (±6.08) of the combination D (2.5% salt, without sugar and pepper, 150 ppm nitrites, temperature fermentation of 10°C). The other combinations showed not significant differences (*p* > 0.05). In the combinations E and F (with the highest salt concentration and the lowest fermentation temperature), no acidification was recorded.

**Table 2:** Fermentation time (FT, day) and fitting parameters of Weibull equation (*Δ*, time to reduce the pH by 1, day; *p*, shape parameter) for the kinetics of acidification.

Strain 69	Δ	*p*	FT
A	9.27 ± 3.00B,C	0.98 ± 0.46B	16.67 ± 5.12B
B	4.14 ± 1.18B	0.93 ± 0.32B	7.97 ± 2.23A
C	7.00 ± 4.84B	0.70 ± 0.54A,B	21.75 ± 5.87B
D	23.05 ± 6.08C	0.46 ± 0.18A	74.75 ± 3.45C
E	-^*^	-	-
F	-^*^	-	-
G	0.52 ± 0.31A	0.34 ± 0.07A	4.93 ± 1.55A
H	6.48 ± 0.43B	1.45 ± 0.38B	10.80 ± 0.56A
Control	5.00 ± 1.29B	1.06 ± 0.50B	9.29 ± 2.33A
Strain 11
A	8.37 ± 1.88B,C	1.32 ± 0.76A,B	12.80 ± 1.80B,C
B	4.89 ± 0.78B	1.00 ± 0.25A,B	8.76 ± 0.87B
C	5.59 ± 2.81B	0.71 ± 0.45A	16.28 ± 4.56C
D	13.21 ± 8.41D	0.73 ± 0.44A	28.18 ± 7.86D
E	-^*^	-	-
F	-^*^	-	-
G	0.33 ± 0.47A	0.31 ± 0.13A	3.88 ± 0.34A
H	6.73 ± 0.32B	1.43 ± 0.25B	11.40 ± 1.12B,C
Control	4.94 ± 1.62B	1.24 ± 0.86A,B	8.42 ± 0.14B
Strain DSM 1055
A	9.07 ± 2.84C	0.89 ± 0.39A,B	17.67 ± 5.67C,D
B	5.27 ± 0.62B	0.98 ± 0.19B	9.72 ± 0.54B,C
C	6.93 ± 4.69C	0.75 ± 0.61A,B	19.87 ± 5.78D
D	7.52 ± 0.76C	3.08 ± 1.48C	8.91 ± 0.57B
E	-	-	-
F	-	-	-
G	1.02 ± 0.83A	0.51 ± 0.17D	5.19 ± 1.01A
H	7.59 ± 0.58C	1.67 ± 0.58C	11.89 ± 0.35C
Control	5.32 ± 0.66B	1.35 ± 0.45C	8.64 ± 0.89B

For each strain and parameter, letters indicate significant differences (one-way ANOVA and Tukey’s test, *p* < 0.05).

* no acidification.

Similar results were also observed for strain 11 with *Δ* values of 0.33 (±0.47) and 13.21 (±8.41) days, in the combinations G and D, respectively. For the reference strain (DSM 1055), the Δ values ranged from a minimum value of 1.02 (±0.83; combination G) to a maximum value of 9.07 (±2.84) days (combination A, 7.5% salt, without sugar, pepper and nitrites, fermentation temperature of 30°C).

Although not having a biological meaning, the “*p*” parameter (shape parameter) represents a useful tool to describe the data trend. In fact, when *p* > 1, the curve has a downward concavity, with an initial phase in which the pH decreases very slowly or does not at all; after this kind of shoulder phase (Geeraerd et al., 2000), a rapid pH decrease is generally observed. On the other hand, when *p* < 1, the curve has an upward concavity, with a rapid initial pH decrease followed by a phase in which the parameter decreases very slowly or does not at all (tail effect). Regardless the strain inoculated, the lowest values of p were recorded for the combination G: in this formulation the kinetic always showed an upward concavity, with *p* values of 0.34 ± 0.07 for strain 69, 0.31 ± 0.13 for strain 11, and 0.51 ± 0.17 for the strain DSM 1055 (Table 2).

Using the fitting parameters of the Weibull equation, the FT (defined as the time in days to attain a pH of 4.4) was also calculated: this pH value is suggested as an essential requirement for food safety, especially to inhibit pathogenic and spoilage bacteria (Riebroy et al., 2008). The FT values are also reported in Table 2. The combination G was confirmed as the best formulation to guarantee the fastest acidification, with the lowest FT recorded, i.e., 4.93, 3.88, and 5.19 days for the kinetics by *L. plantarum* 69, *L. plantarum* 11, and *L. plantarum* 1055, respectively.

In order to evaluate the effects of the different studied variables [concentrations of sugar (Z), salt (S), nitrites (N), white pepper (P), and temperature (T)] on the FT, the FT values were used as input values for a multiple regression analysis: Table 3 shows the standardized effects recovered.

**Table 3:** Standardized effects of temperature, sugar, salt, pepper, and nitrite on the fermentation time.

	69	11	DSM 1055
Temperature	−6.36	−4.72	-
Sugar	−4.13	-	-
Salt	-	-	-
Nitrites	3.32	-	-
Pepper	-	-	-
Curvature	−4.00	−3.05	-
R^2^_ad_	0.896	0.797	-
MS	58.63	25.45	

MS, mean square residual.

**Figure 1:**
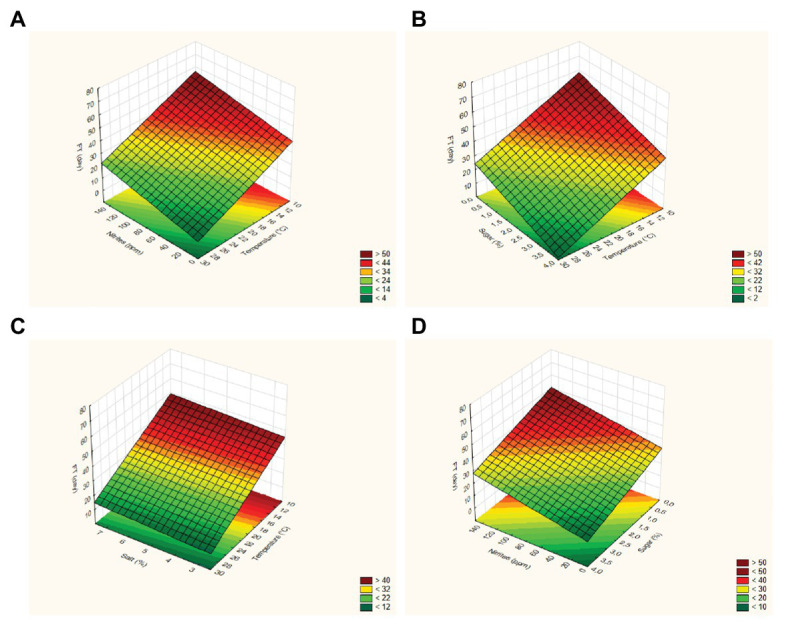
Surface plots for the effects of the interactions [Temperature] / [Nitrites] (part **A**), [Temperature] / [Sugar] (part **B**), [Temperature] / [Salt] (part **C**), and [Sugar] / [Nitrites] (part **D**) on the FT (Fermentation time) values calculated for *Lactiplantibacillus plantarum* 69.

**Figure 2:**
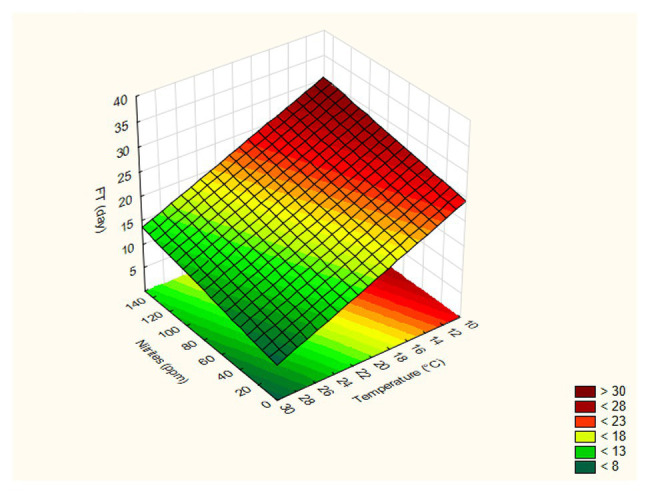
Surface plot for the effects of the interactions [Temperature] / [Nitrites] on the FT (Fermentation time) values calculated for *L. plantarum* 11.

The FT of strain 69 was strongly influenced by temperature and sucrose concentration, while the effects of pepper and salt were not significant (*p* > 0.05); the adjusted regression coefficient was 0.896, while the mean square residual was 58.63, thus suggesting an adequate fit of the model. In addition, the fitting estimates a curvature response with possible slight interactive terms; however, the design used in the present study could be used to estimate both interactive and quadratic response otherwise confounding effects and artifacts could be pointed out. A surface plot can be advantageously used to highlight these effects: in particular, these graphs can be obtained by plotting the FT as a function of two variables. As an example, Figures 1A–D show the surface plots for the effects of the interactions [T] / [N] (part A), [T] / [Z] (part B), [T] / [S] (part C), and [Z] / [N] (part D) on the FT values calculated for *L. plantarum* 69; as expected, the FT decreased as the process temperature and the sugar concentration increased, recording a minimum value at 30°C and 4% of sugar. At 30°C, the effect of salt was not significant (Figure 1C). For *L. plantarum* 11 (Table 3; Figure 2), only the temperature was significant. Finally, the FT of *L. plantarum* 1055 was not affected by any variable studied (Table 3).

Based on these results, the optimal composition was identified in (g/kg): NaCl, 40; NaNO_2_, 0.10; sucrose, 40; white pepper, 10. The process temperature was set at 30°C. This last parameter, together with the sugar and nitrite concentrations, was chosen on the basis of the results obtained, while the concentrations of salt and white pepper were chosen on the basis of literature data (Adams, 2009).

In the second step, *salami-like* were produced according to the individuated formulation, inoculated with the studied starters (10^7^ cfu/g), stuffed into a natural casing and fermented at 30°C for 7 days. The initial pH of fish mixture was 6.50: as the fermentation advanced, all inoculated samples exhibited lower pH than the control. In sausages inoculated with starter strains, the acidification started immediately after the inoculum and the minimum pH (4.3) was found after only 2 days, against the 6 days required to attain the same acidification level in control sample. During 21 days-storage, aerobic microflora, PSE, E, and LAB populations were assessed. In the control, the initial LAB count was low (3.0 log cfu/g), whereas LAB counts of 6.3–7.2 log cfu/g were recovered in all inoculated samples. After 5 days, LAB attained a level of ca. 8.5 log cfu/g. Aerobic bacteria trends were similar to those observed for LAB.

*Pseudomonadaceae* and *Enterobacteriaceae* were ca. 3 log cfu/g in all samples for the entire storage, without significant differences between inoculated samples and the control (data not shown). pH and a_w_ values did not undergo significant changes, recording values about 4.25–4.30 and 0.96–0.97, respectively (data not shown).

**Figure 3:**
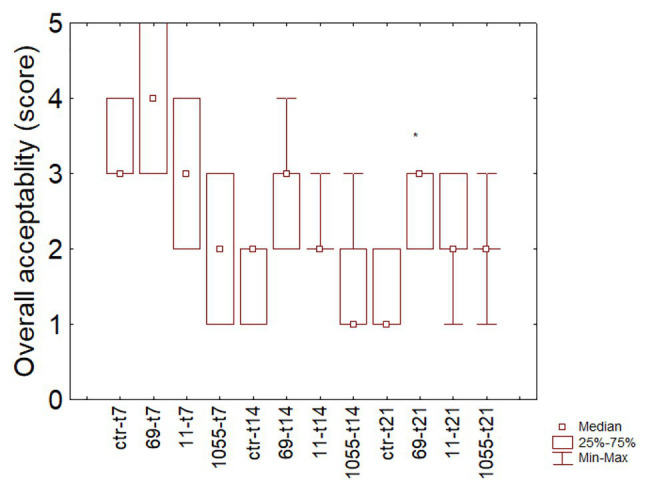
Box-whisker for the sensory scores of fermented sausages during the refrigerated storage. 11, 69, 1055, products inoculated; ctr, control. The numbers after the isolate indicate the time of analysis (7, 14, or 21 days).

Color and odor were the limiting factors for the overall acceptability (data not shown). Figure 3 shows the box-whisker plot for this parameter during the storage: after 21 days, one-way ANOVA revealed that sensory scores of the sausages inoculated with *L. plantarum* 69 were significantly higher than other samples that attained a value nearby the non-acceptability limit (2).

## Discussion

The fermentation of fish for human consumptions has several benefits: first, it is a low cost convenient preservation method, and then it improves the nutritional value and the digestibility or the raw material. Most of fermented fish products are still produced according to local tradition and preferences, leaving the fermentation process to indigenous microbes from the environmental or raw material (Adams, 2009). If referred to small-scale processing units using traditional techniques, even if a high level of product variability is observed, spontaneous fermentation could be still accepted. However, considering globalization and market opening, these products are now widespread also in Europe and North America, and inoculation with starter cultures has become increasingly necessary to improve and stabilize the quality of fermented fish (Nie et al., 2014). Fermented fish products are generally prepared by mixing the fish substrate with salt and other ingredients, such as carbohydrates, spices, or other additives; the product is fermented at various temperatures (from 10 to 45°C) for variable periods (1 week or different months). As for other fermented food, the primary role of starter cultures is to decrease the pH (<4.5), in order to create the conditions which, together with other variables (namely salt and spices) could inhibit the growth undesirable bacteria and extend shelf-life (Adams, 2009). Consequently, the first step of this study was aimed to individuate both the optimal formulation of the fermented product, in terms of salt, sucrose, nitrates and spices concentrations, and the fermentation temperature, since a faster acidification remains the main factor able to guarantee the success of the transformation. For each of the studied strains, the results obtained highlighted that the lowest fermentation time, i.e., time to attain a safe pH of 4.4 (Riebroy et al., 2008), was attained for a formulation containing 4% of sucrose and 100 ppm of nitrite, while the effects of pepper and salt were not significant. The acidification was also strongly influenced by the temperature, with an optimum at 30°C. If this last effect is not surprising, since the temperature is one of the critical parameters affecting the growth of starters and the production of enzymes during fermentation (Thomas et al., 2013), on the other hand, the not significant effect of salt was not expected. Salt concentration in fermented products often ranges from 1 to 10% (w/w); high salt concentrations generally were recommended to better inhibit the growth of spoilage microorganisms, but it should also be considered that a high salt concentration could reduce protease activity (Kim et al., 2003; Klomklao et al., 2006), resulting in a longer fermentation. The optimal formulation was chosen by considering the results obtained by the design for sugar (40 g/kg) and nitrite (0.10 g/kg) concentrations, whereas the concentrations of salt was chosen (40 g/kg) by considering that a too high concentration could cause great harm to human health (Alderman, 2000) and a delayed process (Klomklao et al., 2006). The pepper concentration (10 g/kg) was chosen because of literature data (Adams, 2009); in fact, the role of pepper is controversial. Some authors (Ono et al., 2015) reported a promoting effect of black and red pepper on the fermentation of *nukadoko* (a Japanese fermented rice), while other authors (Kang et al., 2015) underlined a negative effect in the fermentation of *kimchi*, a fermented cabbage.

In general, an optimization step is an overly complex phase, since researchers should focus on many variables; in the present study, this problem was solved using the approach of the Design of the Experiments (DoE). This technique allows to designing and planning significant experiments by minimizing the number of the samples to be analyzed (Bevilacqua et al., 2010).

Once individuated the formulation of ingredients and the process temperature (30°C), a validation in real conditions was performed. A process optimization usually involves a model building *in vitro* by laboratory assays followed by a validation in real system, but in this study, both the DoE and the confirmatory assays were conducted *in situ* (in fish).

The results obtained in the second phase highlighted that the starter strains used assured the correct course of fermentation, reduced the fermentation time and ensured a good microbiological, chemico-physical, and sensorial quality of the final product. In particular, the use of autochthonous starters appeared as a precious tool for the production of fermented fish products as also reported by Zeng et al. (2017), Han et al. (2020), Kusmarwati et al. (2020), and Zang et al. (2020). The latters, for example, used two strains of *L. plantarum* and one strain of *Lactococcus lactis* isolated from spontaneously fermented *Yucha*, a traditional Chinese home-made fermented fish product, as starter cultures for the same product and observed a faster acidification and an improvement of flavor and safety. Similarly, a better quality of *peda*, a traditional salted fermented fish product of mackerel fish of Indonesia, was observed during the use of *Leuconostoc mesenteroides* ssp. *cremonis* BN12 as starter culture (Kusmarwati et al., 2020).

## Conclusion

The production of fermented-fish products with the appearance of salami is a complex process; natural fermentation could not assure the basic requirements of such products: low pH and rapid acidification. Thus, the use of starter cultures is advisable, mainly wild strains, as shown by the performances of *L. plantarum* 11 and 69.

Ingredients and temperature could play a significant role and the results of the first step highlighted the statistical weight of temperature, and nitrites; the use of sugar could increase the acidification rate.

On the other hand, salt and pepper did not exert a significant effect.

The production of a salami-like product through a guided fermentation with selected strains of *L. plantarum* assured a good microbiological quality and sensory scores for at least 21 days of refrigerated storage.

In conclusion, the results obtained in this study suggest that fermented food industries could formulate their own starters to standardize the process without affecting the sensorial characteristics of their traditional products.

## Data Availability Statement

The raw data supporting the conclusions of this article will be made available by the authors, without undue reservation.

## Author Contributions

BS, AB, and MC: conceptualization. CA and AB: methodology. AB: software. BS, AR, and DC: investigation. MS and MC: resources. BS and AB: data curation and writing – original draft preparation. All authors contributed to writing – review and editing. MC and AB: supervision. MC: funding acquisition. All authors contributed to the article and approved the submitted version.

### Conflict of Interest

The authors declare that the research was conducted in the absence of any commercial or financial relationships that could be construed as a potential conflict of interest.
